# Morphine induces endocytosis of neuronal μ-opioid receptors through the sustained transfer of Gα subunits to RGSZ2 proteins

**DOI:** 10.1186/1744-8069-3-19

**Published:** 2007-07-17

**Authors:** María Rodríguez-Muñoz, Elena de la Torre-Madrid, Pilar Sánchez-Blázquez, Javier Garzón

**Affiliations:** 1 Neurofarmacología, Instituto de Neurobiología Santiago Ramón y Cajal, Madrid E-28002, Spain

## Abstract

**Background:**

In general, opioids that induce the recycling of μ-opioid receptors (MORs) promote little desensitization, although morphine is one exception to this rule. While morphine fails to provoke significant internalization of MORs in cultured cells, it does stimulate profound desensitization. In contrast, morphine does promote some internalization of MORs in neurons although this does not prevent this opioid from inducing strong antinociceptive tolerance.

**Results:**

In neurons, morphine stimulates the long-lasting transfer of MOR-activated Gα subunits to proteins of the RGS-R7 and RGS-Rz subfamilies. We investigated the influence of this regulatory process on the capacity of morphine to promote desensitization and its association with MOR recycling in the mature nervous system. In parallel, we also studied the effects of [D-Ala^2^, *N*-MePhe^4^, Gly-ol^5^] encephalin (DAMGO), a potent inducer of MOR internalization that promotes little tolerance. We observed that the initial exposure to icv morphine caused no significant internalization of MORs but rather, a fraction of the Gα subunits was stably transferred to RGS proteins in a time-dependent manner. As a result, the antinociception produced by a second dose of morphine administered 6 h after the first was weaker. However, this opioid now stimulated the phosphorylation, internalization and recycling of MORs, and further exposure to morphine promoted little tolerance to this moderate antinociception. In contrast, the initial dose of DAMGO stimulated intense phosphorylation and internalization of the MORs associated with a transient transfer of Gα subunits to the RGS proteins, recovering MOR control shortly after the effects of the opioid had ceased. Accordingly, the recycled MORs re-established their association with G proteins and the neurons were rapidly resensitized to DAMGO.

**Conclusion:**

In the nervous system, morphine induces a strong desensitization before promoting the phosphorylation and recycling of MORs. The long-term sequestering of morphine-activated Gα subunits by certain RGS proteins reduces the responses to this opioid in neurons. This phenomenon probably increases free Gβγ dimers in the receptor environment and leads to GRK phosphorylation and internalization of the MORs. Although, the internalization of the MORs permits the transfer of opioid-activated Gα subunits to the RGSZ2 proteins, it interferes with the stabilization of this regulatory process and recycled MORs recover the control on these Gα subunits and opioid tolerance develops slowly.

## Background

In the nervous system, G protein-coupled Mu-opioid receptors (MORs) drive the initial steps of both the positive effects of opioids (i.e. relief of intense inflammatory pain) and their addictive effects. A desensitization to morphine that last for several days can occur within hours of administering an appropriate single dose [1] and this is accompanied by some degree of physical dependence [2]. Both single-dose tolerance and that promoted by repeated exposure to morphine seem to share some certain molecular mechanisms. Indeed, both situations can be modulated by similar pharmacological treatments [3].

The inactivation of G protein-coupled receptors (GPCRs) commences with the activation of G proteins upon agonist binding, which in turn produces the segregation of GαGTP subunits from the Gβγ dimers. The increased pool of free Gβγ dimers facilitates their binding to the G protein-coupled receptor kinases (GRK) and hence, the interaction between these kinases and the receptors. In this way, the agonist-bound receptors become a GRK substrate, leading to the phosphorylation of critical cytosolic serine/threonine residues in the receptor. This modification enables β-arrestin to bind to these residues if the agonist remains bound to the receptor [4], setting in motion an endocytic process. Recycling of these internalized receptors to the plasma membrane must occur for the response to agonists to be more rapidly recovered [5]. However, the proteolytic degradation of the endocytosed receptors in lysosomes promotes the down-regulation of the number of surface receptors and brings about a decreased response to the agonist [6].

The phosphorylation of serine 375 in the C terminus of the MOR accompanies the agonist-driven internalization process [7,8]. Although the endocytosed MORs can be sorted into lysosomes, the majority recycle rapidly to the plasma membrane through a signal-dependent process [9]. Interestingly, the efficiency of opioid agonists to stimulate MOR endocytosis differs and this is related to their capacity to promote GRK-dependent phosphorylation of cytosolic residues in the MOR [10,11]. It is believed that morphine induces a high degree of desensitization because it fails to provoke significant phosphorylation and internalization of the MORs [12]. Therefore, opioid agonists that efficiently promote MOR endocytosis would not be associated with high opioid tolerance [13].

It is evident that studies on cells have revealed some critical mechanisms that control the activity of cell surface MORs. However, there is still limited information on the molecular processes that are involved in regulating MORs in the mature nervous system. In this respect, opioid agonists such as etorphine and DAMGO have been shown through immunofluorescence techniques to produce MOR internalization in brain, spinal cord and dorsal root ganglia neurons [14-16]. Notably, and in contrast to what is observed in cultured cells, morphine produces some membrane trafficking of the MORs in dendrites of nucleus accumbens neurons and more extensive MOR internalization in embryonic striatal neurons and ganglia neurons [17-19]. Therefore, although the essential mechanisms of MOR regulation established in cultured cells could apply to neurons, these highly specialized cells also have their own rules to control GPCR function. For example, the expression of certain RGS proteins such as members of RGSZ1, RGSZ2, RGS-R7 subfamily, and of Gαz subunits, is virtually restricted to nervous tissue, and these proteins certainly influence the regulation of neural MORs [20].

We set out here to evaluate the implication of the phosphorylation, internalization and recycling of MORs on the desensitizing capacity of morphine and DAMGO in the murine nervous system. We show that tolerance to intracerebroventricular (icv) morphine was induced by the stable transfer of part of the MOR-activated Gα subunits to RGS proteins of the R7 and Rz subfamilies [21,22], thereby increasing the pool of free Gβγ dimers in the receptor environment. Afterwards, subsequent doses or prolonged exposure to this opioid promoted the GRK phosphorylation of MORs and their internalization and recycling. In these circumstances, the effects that remain after the first dose now desensitized at a much slower rate. DAMGO evaded the first part of this process directly producing the efficient Ser375 phosphorylation and recycling of MORs, which was accompanied by low tolerance to its effects. However, repeated exposure to these opioids led to the incomplete recycling of the MORs and strong tolerance developed.

## Results

### Desensitizing capacity of morphine and how it is influenced by the interval between doses

The alleviation of intense inflammatory pain is the most positive effect of opioids and therefore, we analyzed the development of antinociceptive tolerance in the light of the changes that affect the MORs. Upon icv administration, opioids gain access to periventricular areas implicated in the control of ascending pro-nociceptive information. The analgesic test involves the application of a noxious thermal stimulus to promote a flick of the mouse tail, and the administration of analgesic drugs increases the time that elapses between these two events. Whereas, this motor response is still observed in the intercollicular decerebrated animal, stimulus of much higher intensity are needed to evoke this behavior in the spinal animal. Therefore, this response comprises a spinal reflex which is under a facilitatory drive from the brain stem and the MORs in the periaqueductal grey matter (PAG) play an important role in the antinociceptive effects of opioids administered by the icv route [23].

**Figure 1:**
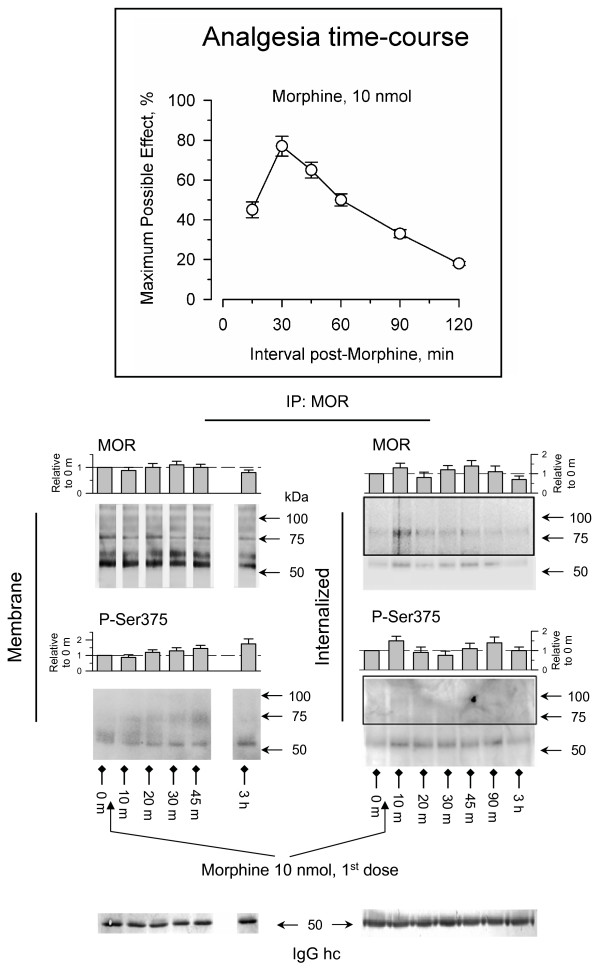
**Regulation of neural MORs by icv administration of an analgesic dose of morphine**. Insert: The mice were icv-injected with 10 nmol morphine and antinociception was determined by the warm water (52°C) tail-flick test at various time intervals post-injection. Antinociception was expressed as a percentage of the maximum possible effect after setting a cut-off time of 10 seconds. The values shown are the mean ± SEM from groups of 10–15 mice. Effect of morphine treatment on internalization and phosphorylation of the C terminal Ser375 of MOR. The PAG synaptosomes (P2) and supernatant (S3) were obtained at various intervals post-morphine administration. For each time point studied the PAG structures from 6 to 8 mice were pooled. To reduce the risk of interference with signals from proteins other than the MORs, the study of these receptors and their Ser375 phosphorylation was performed by immunoprecipitation after releasing the associated proteins by SDS solubilization (denaturing conditions, see Methods). In order to detect additional protein bands, the areas inside the rectangles were overexposed. The densitometric immunosignals associated with the 55–65 kDa band (average optical density of the pixels within the object area/mm2; Quantity One Software, BioRad) were normalized to those obtained probing the anti-MOR IgGs hc (heavy chain) with the appropriate secondary antibody. These IgGs were detached from the immunoprecipitated MORs and processed in parallel gels/blots (see Methods). Each bar is the mean ± SEM of three assays performed on PAG samples obtained from independent groups of mice. The data are expressed relative to the levels observed for the control group (attributed an arbitrary value of 1).

The analysis of MORs in the PAG reveals a series of isoforms that are produced by the alternative splicing of the murine MOR [24], and also by *N*-glycosylation of these variants [25]. Accordingly, these MORs are generally observed at apparent masses of 50–65 kDa, 80–100 kDa and even higher. In our experimental paradigm, icv administration of 10 nmol morphine produced time-dependent antinociception that peaked 30 min after opioid administration and that reached about 80% of the maximum effect measurable in this test. This analgesia had ceased 3 h after the administration of the opioid (Fig. 1). During the time-course of the initial dose of morphine and beyond, the surface levels of MORs remained practically unchanged and the serine 375 displayed moderate phosphorylation. Accordingly, no substantial internalization of MORs was produced and only a small signal could be immunoprecipitated from the supernatant (Fig. 1).

**Figure 2:**
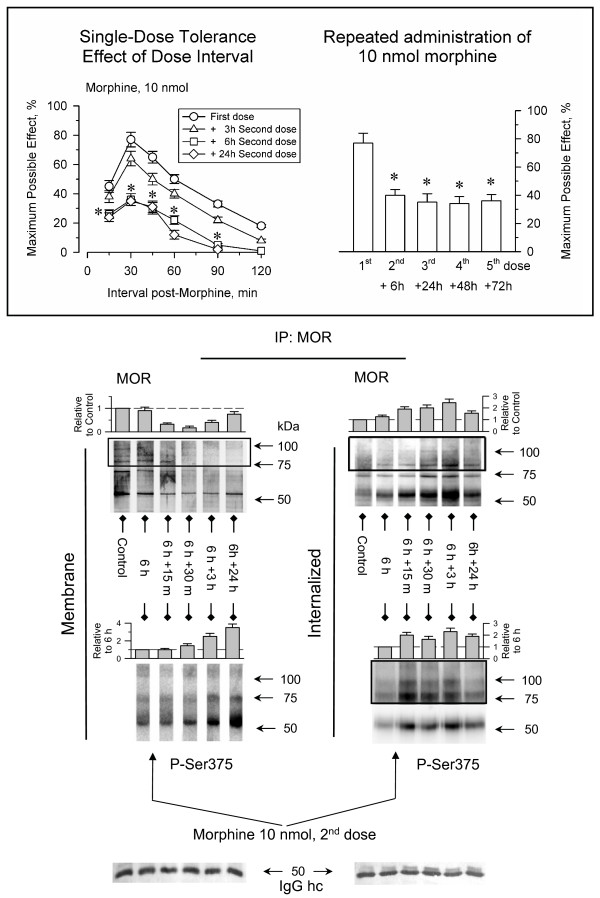
**Single-dose tolerance induced by morphine: influence of the interval between doses**. Insert: *Left panel*, the mice were icv-injected with 10 nmol morphine and after the analgesic effect had ceased, desensitization was evaluated by icv-injection of a second and identical dose of this opioid at different time intervals. *Right panel*, the animals received several icv-injections of 10 nmol morphine spaced 6 h, 24 h, 48 h and 72 h from the first, and antinociception was determined in the tail-flick test at its peak effect 30 min after each injection. Values shown are the mean ± SEM from groups of 8–12 mice. *Statistically significant with respect to the control (First dose) group; ANOVA, Student-Newman-Keuls test (SigmaStat, SPSS Science Software, Erkrath, Germany). Significance was set at *P *< 0.05. The internalization and Ser375 phosphorylation of the MORs was studied after administration of a second dose of 10 nmol morphine 6 h after the first. For further details see Fig. 1 and Results.

The induction of single-dose tolerance is a time-dependent phenomenon that develops after the first exposure of the animals to the opioids and it can be impaired by inhibitors of protein synthesis [26]. Thus, we determined the interval between morphine administrations required to detect this tolerance. When the initial dose of 10 nmol morphine was repeated 3 h after the first dose, the analgesic effect was comparable to that of the first [27]. However, at intervals of 6 h or 24 h the analgesia produced by morphine was much weaker (Fig. 2). The antinociceptive potency of the initial dose of morphine was recovered after 4 to 5 days [28]. Interestingly, this time frame is that required to recover the analgesic effects from the action of β-funaltrexamine, an irreversible antagonist of MORs [29]. These observations suggest that new synthesis of MORs is required to overcome the tolerance that follows an acute dose of morphine.

### Phosphorylation and internalization of MORs stimulated by two consecutive administrations of morphine

When a second dose of morphine was icv-injected 6 h after the first, antinociceptive tolerance was accompanied by a reduction in the amount of MORs in the membrane coupled to an increase in the proportion of intracellular receptors. Moreover, whilst the first dose of morphine caused moderate serine375 phosphorylation of MORs, this second dose promoted notable phosphorylation of the receptors that could be detected both at the surface as well as in the internalized MORs (Fig. 2). The Ser-phosphorylated MORs could be detected in the plasma membrane even 24 h after of administration of this second dose of morphine. Therefore, the repeated administration of morphine promoted both phosphorylation and internalization of MORs, and now the decrease of surface MORs correlated with an increase in the internalized receptors. After this second dose of the opioid, further doses morphine spaced at intervals of 24 h produced comparable peak analgesic effects. Thus, after promoting a high tolerance then morphine is able to support the recycling of the MORs, and then tolerance to this abated effect develops at a slow rate. However, the recycling of MORs receptors seems to be only partial, and reductions in the intervals between consecutive doses could augment antinociceptive desensitization.

### DAMGO promotes Ser375 phosphorylation and internalization of MORs in mature neurons

**Figure 3:**
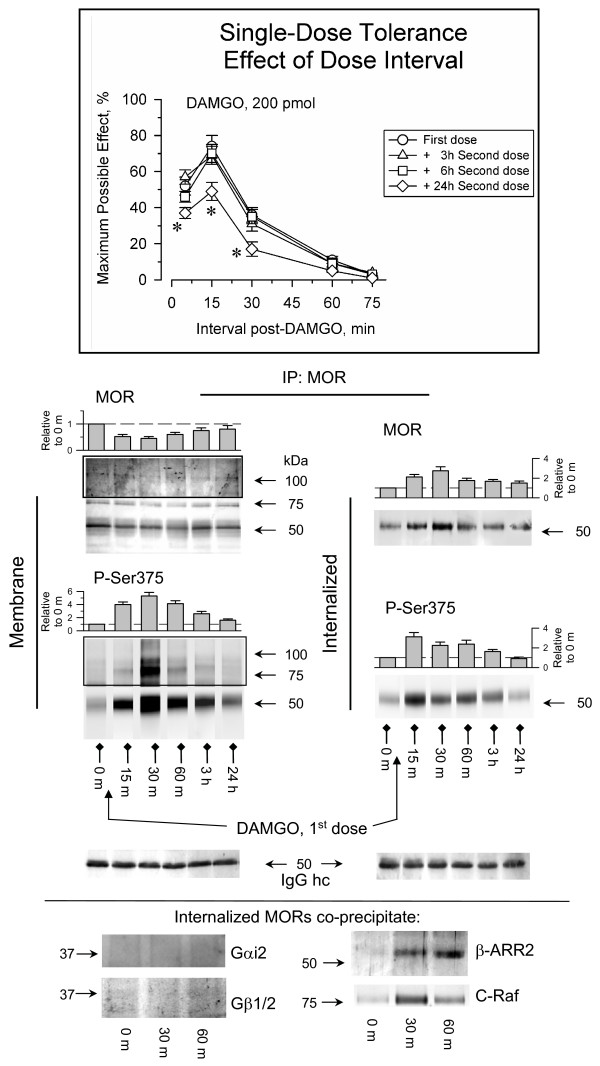
**Regulation of neural MOR phosphorylation and internalization by DAMGO**. Insert: The mice were icv-injected with 200 pmol DAMGO and antinociception was determined by the warm water (52°C) tail-flick test at various time intervals post-injection. The desensitization produced by the priming dose of DAMGO was evaluated injecting a second and identical dose of this opioid at various intervals after the first dose. *Statistically significant with respect to the control (First dose) group; ANOVA, Student-Newman-Keuls test (SigmaStat, SPSS Science Software, Erkrath, Germany). Significance was set at *P *< 0.05. Details as in Figs. 1 & 2. The phosphorylation and internalization of MORs was also evaluated after administering the first dose of DAMGO. The internalized MORs co-precipitated β-arrestin-2 (β-ARR2) and C-Raf but not Gαi2 or Gβ1/2 proteins. For further details see Fig. 1 and Results.

The maximal effect of an icv dose of 200 pmol DAMGO was similar to that with 10 nmol morphine, producing about 80% of the MPE. However, the analgesic potency of a second dose of DAMGO was maintained when injected 6 h after the first. Only when this interval increased to 24 h was a moderate decrease in the effects of this dose observed (Fig. 3). In contrast to morphine, the initial dose of DAMGO stimulated strong Ser375 phosphorylation and internalization of MORs in PAG neurons. However, an important part of the internalized receptor recycled to the plasma membrane within 3 h of the administration of DAMGO when its analgesic effects had ceased (Fig. 3). While the internalized MORs were no associated with Gαi2 or Gβ1/2 subunits, they did co-precipitate with β-arrestin2 and C-Raf. This observation suggests that the internalized MORs regulate β-arrestin2-dependent pathways in these neurons [4]. Thus, our results are in agreement with those describing some internalization of MORs in the adult rodent brain as a consequence of systemic administration of acute doses of etorphine (DAMGO) and the failure of an acute morphine administration to provoke a detectable loss of these receptors [14]. Moreover, our observations in PAG neurons are comparable with those with DAMGO in HEK 293 cells expressing MORs. In this model DAMGO produces the robust Ser375 phosphorylation and internalization of MORs [7,8], and its removal facilitated the recycling of MORs to the plasma membrane and the recovery of the sensitivity to the agonist.

### Desensitization of MORs after repeated administrations of morphine and DAMGO

**Figure 4:**
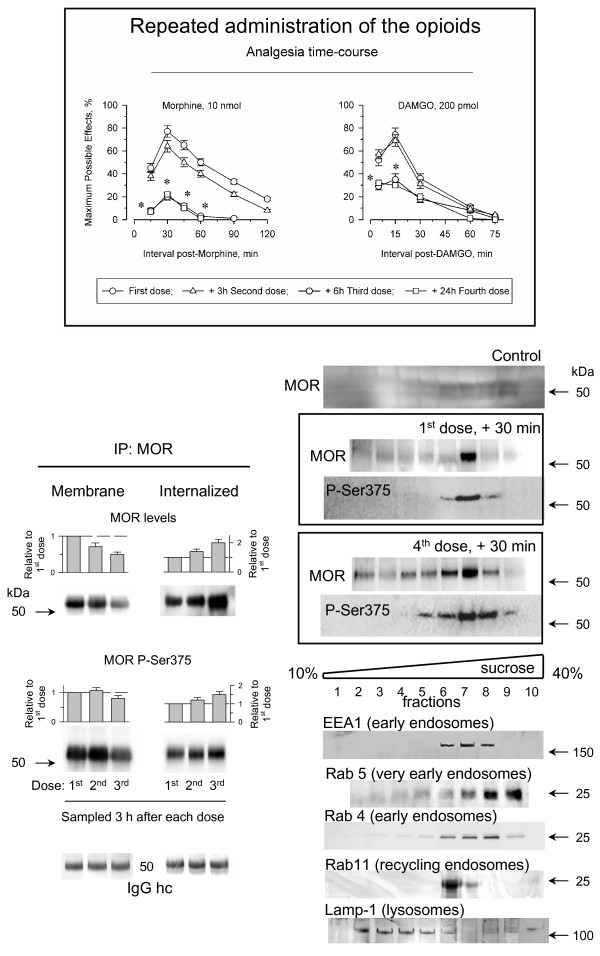
**The repeated administration of DAMGO promotes incomplete recycling of internalized MORs**. Insert: Groups of mice were icv-injected with three successive doses of 10 nmol morphine or 200 pmol DAMGO spaced 3 h apart, plus a fourth dose given 24 h after the first. Antinociception was determined by the warm water (52°C) tail-flick test at various time intervals post-injection. Details as in the Figs. 1 & 2. The mice were killed 3 h after the first, the second or the third dose of DAMGO and the MORs were immunoprecipitated under denaturing conditions from the P2 (membrane) and S3 (internalized) preparations. Mice that had received four doses of DAMGO were killed 30 min later and the PAG S3 fraction was subjected to subcellular fractionation and the presence of MOR was determined. Subcellular markers: EEA1 (early endosome antigen 1; BD 610456), Rab4 (BD 610888), Rab5 (BD 610281), Rab 11 (BD 610656), Lamp-1 (lysosomal-associated membrane protein 1; BD 611043).For further details see Methods.

The differences observed in the capacity of opioids to produce desensitization have been attributed to their intrinsic efficacy. This idea assumes that DAMGO activates only a fraction of the MORs required for morphine to produce comparable analgesic effects, thereby promoting much lower tolerance. However, DAMGO could also stimulate antinociceptive tolerance by reducing the number of plasma membrane MORs. To test this possibility we analyzed the capacity of DAMGO and morphine to produce tolerance when a more demanding administration protocol was used. Morphine and related opioids provoke a profound desensitization of their analgesic effects when three consecutive doses are icv-injected into mice at 3 h intervals [27]. Unfortunately, this protocol also produced desensitization to the analgesic effects of DAMGO, and the second and third consecutive doses increased the loss of surface receptors, thereby producing desensitization to the analgesic response to this opioid (Fig. 4). Indeed, the successive administrations of morphine also slowly diminished the density of cell surface MORs (not shown). The decrease in surface MORs correlated with an increase in the internalized MORs. While the MORs recycle rapidly, a proportion of the internalized MORs undergoes endocytic sorting to lysosomes and thus, proteolytic degradation [8,9]. The subcellular fractionation of PAG synaptosomes revealed that 30 min after the initial icv-injection of DAMGO, the internalized MORs were detected in the early endosome and the recycling endosome fractions. However, after four consecutive doses administered at 3 h intervals, the internalized receptors accumulated and could be also detected in the late endosome/lysosome fraction for their destruction (Fig. 4).

**Figure 5:**
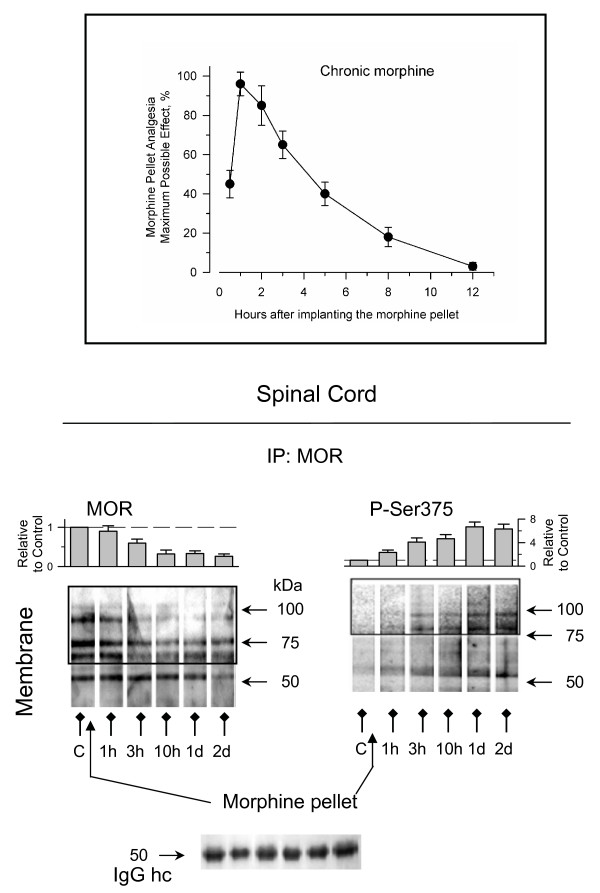
**Development of tolerance to sustained morphine treatment: changes in phosphorylation and surface presence of spinal MORs**. Insert: Animals were subcutaneously implanted with an oily morphine suspension (time zero). Subsequently, the development of tolerance was monitored at various intervals post-opioid administration by measuring the analgesia produced by the release of the opioid. Groups of 10 mice were sacrificed at different intervals and the dorsal horns of the cervical-dorsal spinal cords were removed. To analyze the phosphorylation and presence of MORs in the plasma membrane, the immunoprecipitation was performed under denaturing conditions. For every post-opioid interval analyzed, densitometric signals associated with 55–65, 70–75, and 90–100 kDa were pooled and normalized to those obtained probing the anti-MOR IgGs (heavy chain). The assay was repeated twice and the results were comparable. Further details as in Fig. 1.

The MORs in the spinal cord play an important role in development of tolerance induced by systemically administered opioids. Thus, we analyzed these receptors during the development of tolerance to morphine administered subcutaneously by implantation of oily morphine pellets. The morphine released from this suspension reaches levels of 10–13 nmol per mL of serum and of about 10 nmol per g of wet brain between 3 h and 12 h [30]. In the hour that followed the implantation of the morphine suspension, the mice exhibited an analgesic response that reached the test cut-off time of 10 s. Subsequently, the mice developed rapid tolerance to this effect which was almost absent 12 h later (Fig. 5). The continuous administration of morphine increased both Ser375-phosphorylation and loss of surface MORs in the dorsal horn of the spinal cord. These changes were rapidly observed after the chronic morphine treatment commenced and persisted over the following 2 days. The reductions were more intense for the MORs that corresponded to protein bands of 75 and 100 kDa. It has been reported that these MOR species are rapidly degraded by the proteosome in HEK 293 cells [31] and our observations suggest a similar situation in spinal neurons. Therefore, the continuous presence of morphine in the receptor environment rapidly shifted the system from the uncoupling of the MORs from the regulated Gα subunits to the phase where GRKs gain access to MORs and promote Ser375 phosphorylation, resulting in the internalization of these receptors. This situation would be comparable to the internalization of MORs observed in the presence of elevated concentrations of morphine in dissociated primary cultures of rat embryonic striatal neurons [18] or mouse dorsal root ganglia neurons [19].

### The coupling of plasma membrane MORs to G proteins reduces tolerance to opioid effects

**Figure 6:**
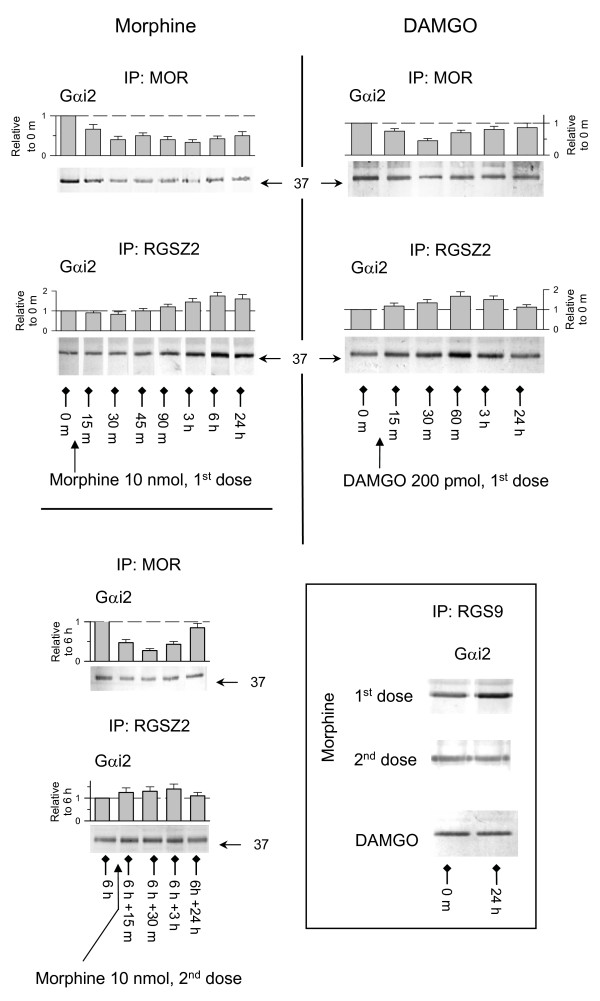
**Opioid-induced transfer of Gα subunits to RGSZ2 and RGS9 proteins**. Groups of 6 to 8 mice, icv-injected with 10 nmol morphine or 100 pmol DAMGO, were sacrificed at different intervals post-opioid administration. Their PAG P2 fractions were then obtained and solubilized under nondenaturing conditions. The MOR, RGSZ2 and RGS9 proteins were immunoprecipitated with specific antibodies from different aliquots of the same solubilized material. The presence of Gαi2 subunits was then analyzed in Western blots in which equal loading was verified by probing anti-MOR or anti-RGSZ2 IgGs in parallel blots using the same immunoprecipitated material. The data corresponding to the co-precipitation of MORs or RGSZ2 with Gαi2 subunits were then normalized and are shown as the mean ± SEM from three determinations (two for RGS9 and Gαi2) performed in different PAG samples.

**Figure 7:**
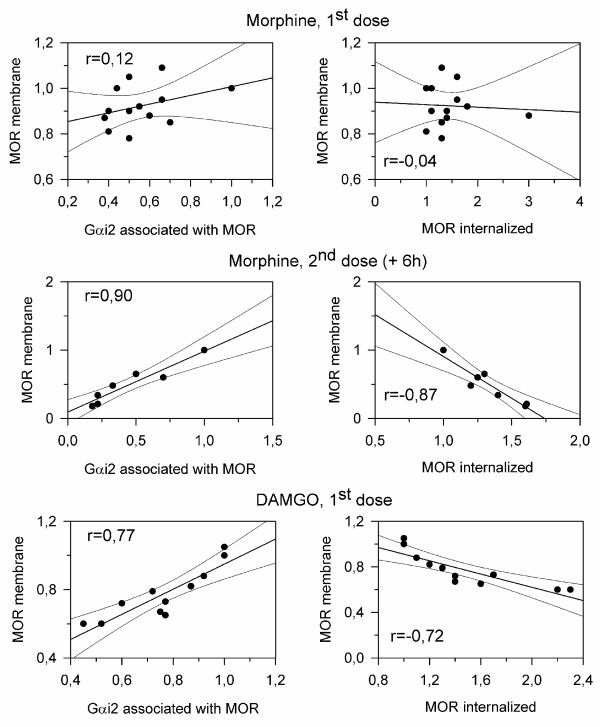
**The coupling of plasma membrane MORs to G proteins reduces tolerance to opioids**. The mice were icv-injected with one or two doses of 10 nmol morphine spaced 6 h apart, or they received a single icv dose of 200 pmol DAMGO. Groups of mice that had received the same opioid treatment were sacrificed at various intervals post-opioid administration. The control mice received icv saline instead of the opioid treatment. The PAG synaptosomes (P2) and supernatant (S3) were obtained and the variations in the surface and internalized MORs (representative data in Figs 1-3), and in the association of surface MORs with Gαi2 subunits, was analyzed (see data in Fig. 6). The densitometric signals corresponding to MORs and the associated Gαi2 subunits that were observed in PAG from control mice injected with saline alone were attributed an arbitrary value of 1. The MOR and Gαi2 values corresponding to mice killed at the post-opioid intervals studied were then normalized to the levels observed for the controls. After normalization of the data, the levels of surface MORs observed at the different post-opioid intervals were correlated with the co-precipitation with Gαi2 subunits, as well as with the levels of internalized MORs. Regression lines, regression coefficients and their confidence intervals of 95% are shown (Sigmaplot v10/Sigmastat v 3.5). The data were pooled from two independent assays.

The initial dose of morphine promoted moderate phosphorylation of Ser375 and little or no internalization of the MORs. However, it did alter the association of plasma membrane MORs with Gα subunits ([21], present work). Notably, the co-precipitation of these receptors with Gαi2 subunits was greatly diminished and only partially recovered 24 h later. Thus, the morphine-activated Gα subunits seem to have been permanently transferred to other compartment. Here, the RGS9 and RGSZ2 play a relevant role (Fig. 6) [21,22]. This reduction of MOR-regulated transduction brought about a substantial decrease in the antinociceptive activity of the subsequent doses of the opioid. Notably, after 6 h the subsequent administration of morphine promoted both phosphorylation and internalization of MORs. A good correlation was now observed between the decreases in surface MORs and the corresponding increase in the internalized pool, as well as with changes in their association with Gαi2 subunits (Figs. 6 &7). In these circumstances, the MORs in the membrane activated the Gα subunits that remained after the first dose of morphine but recovered their control after the effects of morphine had ceased. During the time-course of the effects of DAMGO, the Gα subunits underwent a transient transfer to RGSZ2 proteins and later, the recycled MORs in the plasma membrane recovered control over these G proteins and the response to DAMGO was resensitized (Figs. 3 &6). The results indicate that endocytosis and recycling of MORs diminished the permanent transfer (sequestering) of Gα subunits to RGS proteins, in this way reducing the tolerance to the effects of subsequent administrations of the opioids [11-13,32]. This was observed for DAMGO (Fig. 3) and for a second dose of morphine, which now promoted low tolerance to the effects of additional doses (Fig. 2).

## Discussion

The interaction of morphine with neural MORs is the initial step, both in the development of tolerance to this opioid and towards physical dependence. By analyzing MORs during the time-course of opioid antinociception, new aspects of the mechanisms that control these G-receptors in nervous tissue were revealed. The initial exposure to DAMGO or morphine brought about changes at the MOR level that compared satisfactorily with those described in cultured cells. In both systems, DAMGO produces robust Ser375 phosphorylation and internalization of the MORs whereas in contrast, morphine only weakly induces these processes. In addition, following the removal of DAMGO the MORs recycle back to the cell membrane resensitizing the response to the opioid. However, on removal of morphine the cells remain desensitized and exhibit cross-tolerance to DAMGO. Therefore, while DAMGO produces low tolerance to the effects of subsequent opioid administration, morphine yields a high tolerance. Nevertheless, in mature neurons and in contrast to what might be expected if the MORs were to resensitize on withdrawal of the agonists, a second dose of DAMGO had a weaker analgesic effect after an interval of 24 h. This time-dependent desensitization of MORs was more evident when the effect of the second dose of morphine was studied, decreasing rapidly until the interval between doses reached about 6 h. Longer intervals did not increase desensitization and the analgesic activity of morphine is progressively restored after 3 or 4 days [28]. These observations coincide with the notion that DAMGO and morphine produce low and high tolerance respectively. However, the delayed tolerance to an acute dose of opioid that operates in nervous tissue is known as single-dose tolerance [1], and this phenomenon is probably related to the permanent transfer of Gα subunits to a subset of signaling proteins specific to this tissue.

There is convincing evidence that relates the ability of DAMGO to promote the Ser375 phosphorylation, internalization and recycling of MORs with its weak desensitizing capacity. Accordingly, when morphine promotes Ser-phosphorylation and internalization of MORs in cells [8,11,32], weak MOR desensitization develops [8,13]. In our experimental paradigm, the second dose of 10 nmol morphine spaced 6 h from the first promoted about one third of the analgesic effects of the first dose, coupled with intense phosphorylation and recycling of the MORs. The reduced antinociceptive effects of this second dose of morphine were relatively well reproduced by subsequent administrations of this same dose of morphine but spaced 24 h apart (present work; [33]). Thus, resensitization of MORs in neurons also requires the recovery of active receptors in the cell membrane. This can be achieved by de novo synthesis, although MOR turnover in the brain takes several days [28,29]. Alternatively, and much more rapidly resensitization may occur through the dephosphorylation and recycling of the internalized MORs to the plasma membrane. While the first situation would correspond to the recovery from the first morphine dose, the second applies to the recovery from DAMGO administration or from a second dose of morphine given at least 6 h after the first. Therefore, the use of agonists such as DAMGO could be associated with a reasonable risk of producing tolerance given that the MORs belong to the class of GPCRs that are rapidly dephosphorylated and recycled after internalization, [12,34]. Nevertheless, a fraction of these internalized receptors are sorted to lysosomes and undergo proteolytic degradation [9]. Thus, the repeated administration of DAMGO or morphine could finally desensitize MORs, as observed after administering three consecutives doses of these opioids. It could be argued that agonists that attain their response by activating only a small fraction of MORs would be preferred for the control of severe pain. However, it must be born in mind when used in demanding protocols, these agonists deplete the surface MORs before the novo synthesis can restore the system, which also leads to inescapable desensitization ([2,35,36], present study). Interestingly, even in the demanding protocol used here, the effects that remain after the third dose of morphine or DAMGO were fairly well reproduced by a fourth dose given 18 h later. Obviously, it is difficult to extrapolate this observation to what it is required to effectively drive opioid consumption. However, the biological effects that these opioids conserve after their repeated administration could control physical dependence and therefore, be responsible for the craving behavior.

Morphine is a representative of a particular class of opioid agonists that are useful as analgesics but that are associated with the risk of producing strong tolerance. The limited capacity of morphine to stimulate both Ser375 phosphorylation and MOR internalization could be due to its high off rate from the activated MOR. Thus, morphine will not remain bound to the receptors for long, thereby reducing the probability of GRK phosphorylation and/or the subsequent binding of β-arrestin to the agonist-activated MOR to initiate internalization. The MORs expressed in HEK 293 cells elude internalization upon exposure to morphine, even if the opioid is incubated for long periods of time at high concentrations [32]. The receptors remain at the cell surface and since G protein coupling is essential to increase their affinity towards agonists but not to antagonists, then phosphorylation and uncoupling from G proteins probably desensitizes MORs [8]. In contrast, the MORs present in embryonic cultured neurons were internalized upon incubation with morphine [18]. While an acute dose of morphine produces desensitization without the loss of surface receptors ([14], present study), the administration of subsequent doses or continuous administration promotes the phosphorylation and internalization of MORs. Therefore, these observations again indicate that different processes regulate MOR activity in mature neurons.

One of such process transfers the control of opioid-activated Gα subunits from the MOR to certain RGS proteins. The internalization of the MORs provokes the return of most of these Gα subunits to re-constitute the G proteins and resensitize the response to the agonist when they again come under the control of the recycled receptors. However, long-lasting transfer (sequestering) of MOR-activated Gα subunits occurs when the effects of morphine reach a certain level [21]. This phenomenon is mediated by proteins of the RGS-R7/Rz subfamilies, among which RGS9 and RGSZ2 are particularly relevant [22,33]. This more persistent interaction seems to be facilitated by post-translational modifications of these RGS proteins, permitting them to bind to the activated Gα subunits but precluding their GAP activity on them. Among such modifications, the phosphorylation of serine residues in the RGS domain of RGS-R7 proteins and the ensuing binding to 14-3-3 proteins appear to be highly relevant [21], as does the sumoylation of specific sequences in the RGS of RGS-Rz proteins [22]. The consolidation of this transfer is time-dependent and is probably mediated by the action of certain kinases. Interestingly, PKC has been implicated in MOR desensitization to morphine, but little in the effects of DAMGO [37]. Moreover, the antagonists of NMDA receptors reduce the development of tolerance to morphine antinociception but have little effect on that promoted by DAMGO [38]. Thus, the sequestering of morphine-activated Gα subunits at RGS proteins could involve the activation of glutamate NMDA receptors, probably via PKC [39]. Further efforts will focus on characterizing these mechanisms responsible for the more resolute transfer of morphine-activated Gα subunits to the RGS proteins.

As consequence of impeding the return of GαGDP subunits would be the accumulation of free Gβγ dimers in the environment of the MOR and the improved access of GRKs. Thus, another dose of morphine will promote GRK phosphorylation of the activated MORs. The internalization of MORs produces a reduction in agonist signaling and thus, this RGS-mediated mechanism would exert only a minor effect. Hence, to diminish the signaling of agonists that promote little or no internalization of MORs (e.g. morphine), neural cells would sequester Gα subunits. In this way, the impact of agonist signaling would be reduced and the GRK phosphorylation of MORs would also increase. The influence of such events depends not only on the effect promoted by morphine but also on the interval elapsed after the initial administration of the opioid [21]. This characteristic could explain why delayed tolerance is only observed when a second dose of morphine is injected within a certain time interval. It could also account for the limited desensitization observed for DAMGO when a second dose was administered 24 h after the first, and no before. At this late interval, moderate sequestering of Gα subunits by RGSZ2 proteins could be consolidated and might provoke the reduction in the antinociceptive response to DAMGO. Mice with reduced levels of RGS9 proteins display both an increase in the analgesic effects of morphine and a poorer single-dose tolerance. Therefore, neural MORs can be regulated at the Gα subunit level, as well as through the associated RGS proteins. Hence, opioid resensitization not only requires MOR internalization but also that the recycled receptors recover control of the G proteins. This knowledge can be complemented with the possibility of delaying the development of tolerance, or even rescuing the system, by influencing regulatory mechanisms that only operate in mature neurons and in which a subset of signaling proteins participates.

## Conclusion

This study shows that neural cells have developed specific mechanisms to control GPCR function when the agonists are poor inducers of receptor internalization. Thus, tolerance to morphine in mature neurons develops through a two step process. Firstly, MORs become depleted of Gα subunits and they develop strong antinociceptive tolerance. Subsequently, additional doses of this agonist provoke the phosphorylation and recycling of the MORs, with the consequence that the effects that remain after the first dose now desensitized at a much slower rate. Agonists such as DAMGO that only activate a small fraction of MORs to attain high levels of analgesia could be the rational choice to control of severe pain. However, it must be born in mind that when they are used in demanding protocols, these agonists deplete the surface MORs before the novo synthesis can replenish the system, leading inevitably to desensitization. These findings may be valuable when considering therapies in which rotation of opioids are considered.

## Methods

### Preparation of membranes from neural cells and subcellular fractionation

In these studies, male albino CD-1 mice weighing 22–25 g were used (Charles River, Barcelona, Spain). PAG synaptosomal membranes were obtained from groups of 6 to 10 mice that were sacrificed by decapitation at various intervals after receiving an icv-injection of DAMGO or morphine. The PAGs were collected and homogenized in 10 volumes of 25 mM Tris-HCl (pH 7.4), 1 mM EGTA and 0.32 M sucrose supplemented with a phosphatase inhibitor mixture (Sigma # P2850), H89 (Sigma, B1427) and a protease inhibitor cocktail (Sigma, P8340), that contained 4-(2-aminoethyl)-benzenesulfonyl fluoride (AEBSF), pepstatin A, transepoxysuccinyl-L-leucylamido(4-guanidino)butane (E-64), bebstatin, leupeptin and aprotinin. The homogenate was centrifuged at 1000 *g *for 10 min to remove the nuclear fraction, pellet 1 (P1). The supernatant (S1) was centrifuged at 20000 *g *for 20 min to obtain the crude synaptosomal pellet (P2). The pellet (P2) was resuspended in buffer and centrifuged at 20000 *g *for an additional 20 min, and the final pellet was diluted in Tris buffer supplemented with a mixture of protease inhibitors (0.2 mM phenilmethylsulphonyl fluoride, 2 μg/mL leupeptin and 0.5 μg/mL aprotinin) before aliquoting and freezing. The supernatant (S2) was centrifuged at 105,000 *g *for 1 h to obtain the crude microsomal pellet (P3) (Beckman XL-70 ultracentrifuge, rotor Type 70 ti). The S3 supernatant was concentrated in Amicon Ultra-4 centrifugal filter devices (nominal molecular weigh limit NMWL of 10,000 #UFC8 01024, Millipore Iberica S.A., Madrid, Spain), and it was then loaded on a 10–40% continuous sucrose gradient and centrifuged at 225,000 *g *for 18 h [[40] and references therein]. Ten 4 mL fractions were collected, the proteins concentrated, and the MORs immunoprecipitated and analyzed by Western blotting.

### Immunoprecipitation of MORs and the co-precipitation of signaling proteins

To evaluate the presence of MORs in the plasma membrane and in intracellular structures the existing protein interactions were disrupted under denaturing conditions prior to performing immunoprecipitation. Thus, the PAG synaptosomal membranes (P2) and supernatants (S3) were heated in 40 mM Tris-HCl, 1% SDS buffer for 10 min at 100°C in the presence of 2-mercaptoethanol. This mixture was then cooled to room temperature and the SDS concentration reduced 7-fold by adding octylthioglucoside to a final concentration of 20 mM, and the immunoprecipitation of MOR was then performed with biotinylated IgGs (Pierce #21217 & 21339) as described below.

The co-precipitation of signaling proteins with the MORs, RGSZ2 and RGS9 proteins was performed under non-denaturing conditions. PAG preparations were sonicated (2 cycles of 5 s each in 400 μL of buffer containing 50 mM Tris-HCl (pH 7.5), 50 mM NaCl, 1% Nonidet P-40, and 50 μL protease and phosphatase inhibitors and H89). The supernatant was cleared by incubating with 20 μL of streptavidin-agarose (Sigma S1638) pre-equilibrated for 1 h at 4°C, which was recovered by centrifugation at 3000 *g *for 5 min. The Nonidet P-40 solubilized proteins were incubated overnight at 4°C with about 3 μg of affinity purified biotinylated IgGs directed against the target protein. The immunocomplexes were recovered with streptavidin agarose and the agarose pellets obtained by centrifugation were washed three times, pelleted and resuspended in Nonidet P-40 buffer. The resuspended immunocomplexes were transferred to centrifugal filter devices (5 μm, Amicon Microcon UFC40SV, Millipore) and the Nonidet P-40 buffer was separated from the IgG-agarose complexes by mild centrifugation. This washing step was repeated twice before the agarose-immunocomplexes retained in the filter membrane were heated in Tris-HCl pH 7,5, 1% SDS buffer at 100°C to separate the IgG-agarose from the target proteins. The immunoprecipitated proteins were then recovered by centrifugation and concentrated in centrifugal filter devices (10,000 kDa nominal molecular weight limit, Amicon Microcon YM-10 #42407, Millipore), solubilized in 2 × Laemmli buffer and resolved by SDS-PAGE. The detached anti MOR IgGs retained in the filter membrane were used to normalize the MOR-related signals. This procedure yielded enough protein to load four to six gel lanes. The proteins were transferred to 0.2 μm polyvinylidene difluoride membranes (PVDF; Amersham Biosciences, Spain) and probed with the selected antibodies in DecaProbe chambers (PR 150, Hoefer-AmershamBiosciences, Barcelona, Spain). The procedures used to prepare the PAG enriched synaptosomes, supernatant and the pull-down experiments have been described in detail elsewhere [21,22].

### Detection of signaling proteins

Western blots were probed with a series of antibodies raised against signaling proteins. The blots were probed with affinity purified IgGs: antibodies directed against peptide sequences in the murine MOR1 (diluted 1:1000): NT (DSSAGPGNISDCSDP, residues 2–16 of the extracellular N-terminal [25]), 2EL (TKYRQGSID, 208–216 of the second external loop [25]), anti Gαi2 (1:1000, [25]); anti-β-arrestin2 (1:1000, Calbiochem 178600); anti-C-Raf (1:2000, BD Transduction labs., 610151). Rabbit polyclonal IgGs against phospho-μ-opioid receptor (Ser375) (1:1000, Cell Signaling, 3451) were used to analyze the phosphorylation state of the MORs. All the antibodies were diluted in TBS + 0.05% Tween 20 (TTBS) and incubated with the PVDF membranes for 24 h at 6°C. The primary antibodies were detected with the corresponding secondary antibodies conjugated to horseradish peroxidase diluted 1:10000 in TTBS. Antibody binding was visualized with the ECL+plus Western Blotting Detection System (RPN2132, Amersham Biosciences) and the chemiluminescence was recorded with a ChemiImager IS-5500 (Alpha Innotech, San Leandro, California) equipped with a Peltier cooled CCD camera that provided a real time readout of 30 frames per second (-35°C; high signal-to-noise ratio; dynamic range up of 3.4 OD). Densitometry was performed using Quantity One Software (BioRad) and expressed as the mean ± S.E. of the integrated volume (average optical density of the pixels within the object area/mm2). For each treatment, the assays were typically performed twice or three times on samples obtained from independent groups of mice and the results were comparable. For assays in which the immunoprecipitated protein was modified by the opioid treatment, equal loading was verified and where necessary the signal adjusted using that obtained when detecting the heavy chain of the IgGs used to precipitate the target protein. The IgGs present in the immunoprecipitated samples were detached from the target proteins and processed in parallel gels/blots [21].

### Animals, intracerebroventricular injection and evaluation of antinociception

Male albino CD-1 mice weighing 22–25 g were housed and handled in accordance with the European Community guidelines for the Care and Use of Laboratory Animals (Council Directive 86/609/EEC). Animals were lightly anaesthetized with ether, and the opioids were injected into the lateral ventricle in a volume of 4 μL, as described previously [41]. The response of the animals to nociceptive stimuli was determined by the warm water (52°C) tail-flick test. Latencies in seconds were determined both before treatment (basal latency) and also after the administration of the substance under study (test latency). Baseline latencies ranged from 1.5 to 2.2 seconds and a cut-off time of 10 seconds was allotted to minimize the risk of tissue damage. Antinociception was expressed as the percentage of the maximum possible effect (MPE = 100 × [test latency-baseline latency]/[cut-off time-baseline latency]).

### Induction and assessment of tolerance upon continuous morphine treatment

Groups of 15–20 mice were subcutaneously (sc) implanted with 10 ml/kg body weight of a suspension containing 50% saline (0.9% NaCl in distilled water), 42.5% mineral oil (Sigma #400-5), 7.5% mannide monooleate (Sigma #M-8546), and 0.1 g/ml morphine base. The oily pellet offers an animal model of opioid tolerance-dependence that reproduces the rapid rise of morphine in serum and brain described for hard pellets [30]. Development of tolerance was monitored by measuring the analgesic response promoted by the release of the opioid.

## Abbreviations

MOR : μ-opioid receptor.

DAMGO : [D-Ala^2^, *N*-MePhe^4^, Gly-ol^5^] encephalin.

PAG : periaqueductal grey matter.

RGS : regulator of G-signaling protein.

## Competing interests

The author(s) declare that they have no competing interests.

## Authors' contributions

MRM and ETM performed the molecular studies, and contributed to the analysis and interpretation of the data. PSB designed and performed the behavioral studies. JG conceived the study, participated in its design, and assisted with the data analysis and interpretation. JG and PSB wrote and revised the manuscript. All authors have read and approved the final manuscript.
